# Validation of a leg-mounted pedometer for the measurement of steps in lactating Holstein cows

**DOI:** 10.3168/jdsc.2023-0403

**Published:** 2023-11-04

**Authors:** J.C.S. Marques, T.A. Burnett, J. Denis-Robichaud, A.M.L. Madureira, R.L.A. Cerri

**Affiliations:** 1 Faculty of Land and Food Systems, University of British Columbia, Vancouver V6T 1Z4, Canada; 2 Ridgetown Campus, University of Guelph, Ridgetown, ON, N0P2C0, Canada

## Abstract

The aim of this study was to validate the pedometer AfiAct II (AfiMilk) for the measurement of steps in lactating Holstein cows housed in a freestall design by assessing its agreement with visual observation of step counts. A total of 41 primiparous (n = 12) and multiparous (n = 29) cows were enrolled in the study between August and September 2018. Steps were monitored continuously by the pedometer and visually assessed for a 24-h period using video recordings. Visually observed steps were categorized as walking and stationary steps. The total number of steps taken per cow was calculated using the sum of walking and stationary steps. Unprocessed step count data from the study day were retrieved from the AfiMilk system in time-blocks of approximately 15 min. Repeated measures correlation was used to quantify the association between the pedometer measurements and visual observation of step counts. Nonindependence among observations were accounted adjusting for interindividual (cow) variability with an analysis of covariance. Pearson correlation coefficients (r) were categorized from negligible (0.00–0.30) to very high (0.90–1.00). Bland-Altman plots were created to evaluate the bias between the pedometer and visual observations. A total of 2,261 time-blocks were used in this study with an average (mean ± standard deviation) of 55.14 ± 8.1 time-blocks per cow. A high correlation was found for the evaluation between the pedometer and observed walking steps (r = 0.74; 95% confidence interval [CI] = 0.73–0.76), stationary steps (r = 0.71; 95% CI = 0.69–0.63), and total steps (r = 0.88; 95% CI = 0.87–0.89). The results of the Bland-Altman plot suggested limited bias between the pedometer step counts and visual observation of steps, independent of the type of steps. Numerical differences and several time-block differences outside of the 95% interval of agreement suggested an overestimation of step counts by the pedometer, which increased as the number of steps increased. The pedometer measured, on average, 97.6 ± 118.5 (28%), 249.2 ± 126.2 (125%), and 297.2 ± 205.4 (196%) steps/day more than the visual observed total steps, stationary steps, and walking steps, respectively. Our findings indicate that the pedometer counts all movement in which the pedometer leg is lifted off the floor without distinguishing if there was body movement of the animal.

## Highlights

• The pedometer has a high correlation with visual observation of step counts.
• The pedometer counts all movement in which the pedometer leg is lifted off the floor.
• The pedometer overestimates step counts compared with visual observations.

## Graphical Abstract

**Figure:**
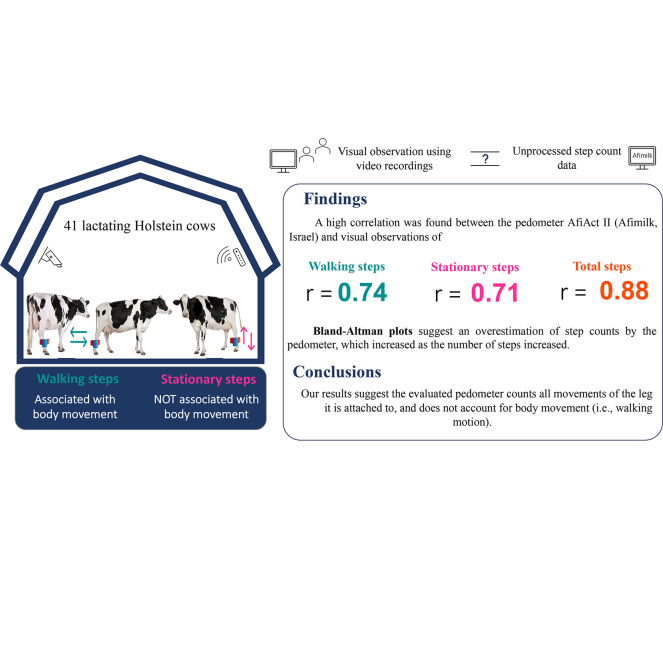
**Summary:** This study was conducted to validate a commercial pedometer for the measurement of steps in pregnant, nonlame, lactating Holstein cows housed in a freestall design. The validation was conducted by assessing the agreement between the technology and visual observation of step counts. Visually observed steps were categorized as walking and stationary steps, and their sum was considered total steps. We demonstrated that the measurements from the commercial pedometer had high agreement with visual observations of step counts whether the steps were measured while the animal was walking or in a stationary position. In addition, an overestimation of step counts by the pedometer in comparison to visual observation was observed.

Automated systems have been developed and implemented on dairy farm operations to remotely collect behavioral data and to decrease the need for visual monitoring (Stygar et al., 2021). A variety of sensors are able to monitor animals individually by measuring physical activity, lying time, rumination, and feeding time (reviewed in Stevenson and Britt, 2017). Data quality from these systems is an important factor as farmers rely on it to make management decisions. In addition, quantitative data from these monitors have the potential to assist on-farm decisions and provide data for digital phenotypes for genetic selection (Cerri et al., 2021).

In the literature, several pedometers have been previously validated for the number of steps taken by dairy cows or calves (Felton et al., 2012; Swartz et al., 2016; Nielsen et al., 2018). Successfully, these studies demonstrated high correlation coefficients (ranging from 0.85 to 0.99) comparing commercial pedometers with visual observation of step counts. However, the pedometer AfiAct II (AfiMilk), while used in many dairy farms, has not been validated for the measurement of steps in lactating Holstein cows. Furthermore, studies validating pedometers for the number of steps taken by the animals (Felton et al., 2012; Nielsen et al., 2018) have not accounted for movement while these steps were taken (i.e., stationary or walking). To date, it is still unclear whether pedometer measurements represent steps while the animal is moving or not, and to which extent misrepresentation of step counts may affect the performance of sensor technologies to detect estrus events or the analysis of quantitative data to define the intensity of estrus.

The objective of this study was to validate a pedometer for the measurement of steps in lactating Holstein cows by assessing its agreement with visual observation. We hypothesized that step counts measured by the pedometer have a high correlation with the visual observation of walking steps, but a low correlation with the visual observation of stationary steps.

This study was conducted in August and September 2018 at the University of British Columbia's Dairy Education and Research Centre, in Agassiz, Canada. All experimental procedures were approved by the Animal Care Committee of the University of British Columbia following protocol A18–0043. Lactating Holstein cows were housed in a deep sand–bedded barn with a freestall design, concrete flooring, and manure scraper. Milking was performed twice daily within a conventional milking parlor (Boumatic Dairy Equipment) located 76 m from experimental pens.

Pregnant non-lame lactating cows housed into experimental pens equipped with cameras and having an AfiAct II pedometer were selected for this study. The device was placed on their back left leg above to the distal expansion of the metatarsal bone at least 4 mo before the expected start date of the study. Two AfiAct readers were located in the freestall barn, one 5 m from the experimental pens and the other 10 m from the milking parlor. Activity data were transmitted from the pedometer to the computer through the readers every 15 min and automatically stored on the AfiFarm SQL database. Unprocessed activity data (step counts) were extracted from the database to Excel (Microsoft Corp.) documents in time-blocks of approximately 15 min.

Visual detection of behaviors and step counts were monitored for a 24-h period using Panasonic WV BP330 cameras positioned approximately 8 m above the experimental pens. The cameras were attached to a video multiplexer (WV-FS416; Panasonic) and a time-lapse recorder (AG-6540p; Panasonic). To facilitate video recording during the night, infrared lights (100 W) were placed approximately 8 m above the pens. The video recordings were accessed using the Remote Viewlog 500 (GV-Remote Viewlog, GeoVision) software. To facilitate identification during video recordings, cows were individually identified with alphanumeric symbols on their backs made with hair dye.

The numbers of steps taken with the leg where the pedometer was placed were counted from the video recordings. Steps were also categorized into walking steps and stationary steps, and their sum was considered total steps. Walking steps were defined as when the cows foot left the ground and the body of the cow moved forward or backward. Stationary steps were defined as when the foot left the ground, but the cow did not move forward or backward. Due to the positioning of the camera, steps were not able to be counted when cows were at the feed bunk or at the time of milking. Presence at the feed bunk began when the cow's ears crossed through the headlocks into the feed bunk and ended when the cow's head crossed back out of the headlocks into the feed alley for more than 30 s. Cows were classified as exiting the pen for milking at the moment the cow's back foot left the pen and classified as returning from the milking parlor the moment that the cow's back foot entered the pen.

One trained individual watched all the recordings used for this study. To determine that the individual was effectively trained and that observations were repeatable, a second observer was trained and watched the same 4 cow-days (96 h) as the first observer. The interobserver reliability was assessed between the 2 observers by calculating the intraclass correlation coefficient (**ICC**) for each behavior described above, including walking and stationary steps. To ensure consistency of observations over time, intraobserver reliability was calculated on 12 h of recording for the main observer after every 120 h of visual readings (equivalent to every 5 cow-days). The ICC were classified as poor (0.00 to 0.40), fair (0.40 to 0.59), good (0.60 to 0.74), or excellent (0.75 to 1.00; Hallgren, 2012). To avoid bias during observation of video recordings, no access to the sensor data was available to the observer until the completion of the visual observations.

A sample size of a minimum of 29 lactating cows was determined following the approach proposed by Borg et al. (2022) for validation studies using a minimum accepted correlation of 0.5 (moderate correlation) and expected correlation of 0.7 (high correlation), an average number of 50 observations per cow, 95% confidence and 80% power. This sample size also accounted for 15% loss to follow-up.

Data preparation and descriptive analyses were carried out using SAS Studio (version 3.8, SAS Institute Inc.). For the step counts data from the pedometer (extracted in 15-min time-blocks), the same 24-h period as the visual detection of behaviors and step counts was selected for the analysis. Time-blocks for which cows were observed (visual observation) as either out of the pen (milking times) or within the feed bunk were removed from the pedometer data. The number of steps per cow during the 24-h period was calculated using the sum of steps measured at each 15-min time-block, for visual observation or pedometer data.

Descriptive analyses were calculated, and continuous variables were assessed for normality and presence of outliers graphically (PROC UNIVARIATE). Bland-Altman plots (Bland and Altman, 1986) were created using Excel to assess the mean difference between the pedometer and visually recorded steps within each 15-min time-block, and per day. The pedometer's measurements was considered accurate if the mean bias from the plots included zero within the 95% interval of agreement.

Repeated measures correlation (rmcorr) was carried out using Rstudio (version 2022.12.0, Rstudio, PBC) to quantify the association between the pedometer measurements and visual observation of walking, stationary, and total step counts. Briefly, the correlation was obtained while adjusting for interindividual (cow) variability with an analysis of covariance (Bakdash and Marusich, 2017). The Pearson correlation coefficient (r) were categorized as negligible (0.00 to 0.30), low (0.30 to 0.50), moderate (0.50 to 0.70), high (0.70 to 0.90), or very high (0.90 to 1.00; Hinkle, 1998). Homoscedasticity and normality of the residuals were assessed graphically.

Linear regression models were used to evaluate the effect of parity, DIM, length of pregnancy, and milk production on the mean difference per day/cow between the pedometer and visually recorded steps (PROC GLM). For all linear regression models, homoscedasticity and normality of the residuals were assessed graphically.

A total of 41 lactating Holstein cows were enrolled into this study given the space availability in the research pen. Cows had an average of 2.7 parity (SD = 1.6; median = 2; range = 1 to 6), 256 DIM (SD = 47; median 257; range = 145 to 373), 142 d pregnant (SD = 36; median = 141, range = 76 to 211), and 12,055 kg of projected mature equivalent 305-d milk production (SD = 2,225; median = 12,075; range = 8,060 to 17,010).

A total of 984 h of video recordings and pedometer data (24-h period per cow) were processed, resulting in a total of 3,728 time-blocks recorded throughout the study with an average (±SD) of 93.2 ± 5.6 time-blocks per cow. However, 1,467 time-blocks (39%) were removed as cows were either out of the pen or within the feed bunk. A total of 2,261 time-blocks were used in this study with an average of 55.1 ± 8.1 time-blocks per cow. The pedometer measured in average 8.2 ± 14.6 steps (range = 0 to 118) per time-block/cow and 449.1 ± 224.7 steps (range = 170 to 1,004) per 24-h period/cow. Visual observation of step counts measured in average 7.1 ± 13.1 (range = 0 to 93) total steps, 3.1 ± 7.7 (range = 0 to 77) walking steps, and 3.9 ± 8.4 (range = 0 to 82) stationary steps per time-block/cow. Per 24-h period, the averages of visually recorded steps were 351.5 ± 172.1 (range = 111 to 978), 199.9 ± 136.2 (range = 34 to 686), 151.9 ± 66.6 (range = 36 to 292) for total, stationary, and walking steps, respectively.

Visual observation had excellent reliability with interobserver ICC ranging from 0.88 to 0.94, and intraobserver ICC ranging from 0.97 to 0.99 (interobserver: stationary steps = 0.88; walking steps = 0.94; total steps = 0.94; intraobserver: stationary steps = 0.97; walking steps = 0.98; total steps = 0.99).

A high correlation was found between the pedometer and observed walking, stationary, and total step counts. Pearson correlation coefficient was highest for the comparison between the pedometer measurements and the observational total number of steps (Figure 1A; r = 0.88; 95% CI = 0.87–0.89), followed by walking steps (Figure 1B; r = 0.74; 95% CI = 0.73–0.76) and stationary steps (Figure 1C; r = 0.71; 95% CI = 0.69–0.63).

**Figure 1:**
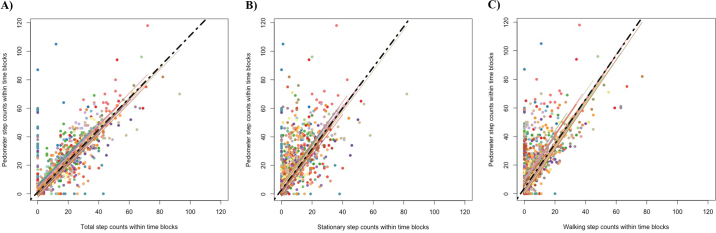
Effect plots from repeated measures correlation models summarizing the correlation between the pedometer step counts and the visual observation of total number of steps (A), stationary steps (B), and walking steps (C). Each data point illustrates a 15-min time-block, and each slope (color) illustrates an individual cow.

The results of the Bland-Altman plot per time-block suggested limited bias between the pedometer step counts and visual observation of steps, independent of the step type (Figure 2). The mean difference (±SD) between the pedometer and the total, stationary, and walking steps were 1.8 (±7.2), 4.5 (±10.9), and 5.4 (±11.1) steps, respectively. Numerical differences and several time-block differences outside of the 95% interval of agreement suggest an overestimation of step counts by the pedometer, in comparison with the visual observation, which increased as the number of steps increased.

**Figure 2:**
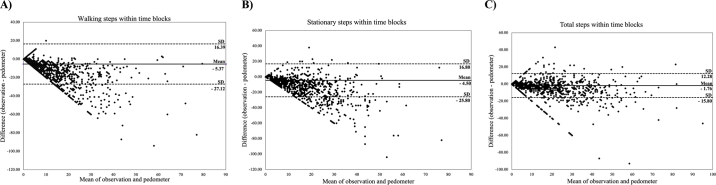
Bland-Altman plot illustrating the agreement between the pedometer and the visually observed walking steps (A), stationary steps (B), and total steps (C) within 15-min time-blocks. The x-axis in all graphs represents the average of the visual observation and the pedometer, and the y-axis represents the difference between the visual observation and the pedometer. Each data point illustrates a 15-min block.

The Bland-Altman plots per day also suggested limited bias of steps count between the pedometer and the visual observation, independently of the type of steps (data not shown). The mean differences (±SD) were 97.6 ± 118.5 (28%), 249.2 ± 126.2 (125%), and 297.2 ± 205.4 (196%) steps between the pedometer and the visually observed total, stationary, and walking steps, respectively. Although several time-block differences were observed within the 95% interval of agreement, numerical differences and the distribution pattern of the differences suggest an overestimation of step counts as the number of steps increases.

The mean difference between the pedometer and visually recorded steps per day was not affected by parity (total steps: *P* = 0.88; walking steps: *P* = 0.33; stationary steps: *P* = 0.45) or DIM (total steps: *P* = 0.57; walking steps: *P* = 0.89; stationary steps: *P* = 0.71). Similarly, independent of the type of steps, the mean difference between the sensor and observed steps was not associated with cow length of pregnancy (total steps: *P* = 0.32; walking steps: *P* = 0.44; stationary steps: *P* = 0.55) or level of milk production (total steps: *P* = 0.37; walking steps: *P* = 0.24; stationary steps: *P* = 0.35).

The correlation coefficient was highest between the pedometer and the total step counts, suggesting that the commercial pedometer counts all movement of the cow's leg lifted off the floor independent of body movement. Swartz et al. (2016) evaluated the same pedometer for the measurement of steps in dairy calves and found a high correlation between the pedometer step counts and visual observation. However, Swartz et al. (2016) defined a step as the movement of the pedometer leg lifted off the floor, not distinguishing according to body movement. A previous version of the pedometer used in this study has been validated for steps activity in dairy cows housed in tiestall design during estrus (Felton et al., 2012). In their study (Felton et al., 2012), high correlations in the comparison between the pedometer and visual observation of steps (not accounting for body movement) were also observed. Although findings from previous validation reports supported the results observed in our study, we believe that the step activity definition used in these studies does not represent true walking activity. This can be a problem as walking and stationary steps do not represent the same behavior. Pedometers are able to detect estrous events by evaluating deviations in step activity comparing the activity value at estrus with activity values of a reference period (Stevenson and Britt, 2017). Therefore, if a cow takes few, or several, stationary steps in a day she could be misclassified by the sensor having an undetected estrous event or a false estrous alert. Furthermore, misrepresentation of step counts could negatively affect research investigation using pedometer data as a measurement of cows' walking activity. For example, studies from our laboratory (Cerri et al., 2021) and others (López-Gatius et al., 2005; Roelofs et al., 2005) have used pedometer data to indicate intensity of estrous expression in dairy cows. However, increased frequency of stationary steps is not a secondary sign of estrus (Roelofs et al., 2010). Thus, the intensity of estrus measured by the commercial pedometer is not a true representation of cows' estrous activity. Future studies should account for body movement when validating pedometers for the measurement of steps in dairy cows.

The total number of steps per time-block averaged between 7 to 8 steps, depending on the type of tool used to measure step activity. However, in other studies (Mattachini et al., 2013; Black and Krawczel, 2016), the absolute number of steps taken by dairy cows housed in freestalls varied from 1,300 to 2,000 steps per day (approximately 13 to 20 steps/15-min). The lower average of step counts found in this study may be due to the removal of the time-blocks where cows were either out of the pen or within the feed bunk. It is possible to hypothesize that the removal of these times, mainly milking times due to the distance from the experimental pens to the milking parlor, may be reducing the average of step counts found. Furthermore, our results demonstrated greater absolute number of stationary steps per day compared with walking steps. Although other validation studies have not accounted for body movement while measuring step activity of dairy cows, other studies (Rajapaksha and Tucker, 2014, 2015) have reported step counts taken by dairy cows while standing on different floor surfaces. In these studies (Rajapaksha and Tucker, 2014, 2015), cows standing on a concrete floor (similar to freestall) were reported to take on average 1.5 stationary steps/minute (approximately 22 steps/15 min). In this context, we hypothesize that the number of stationary steps found in our study is possibly normal and the numerical difference found between walking and stationary steps is perhaps associated with the removal of times with high walking step activity.

Plots from repeated measures correlation and Bland-Altman plots demonstrated a variation in the pedometer performance between cows. Given that cow parity, length of gestation, DIM, and their level of milk production were not associated with changes in the pedometer performance, we believe that individual characteristics do not affect the ability of the pedometer to measure step activity in dairy cows. Perhaps the technical properties of the sensor, such as within- and between-instrument variation, could partially explain the variation seen in our study. However, to date not all of the properties of private technologies are publicly known (Gengler, 2019) due to privacy and security features deployed by manufacturers (Wolfert et al., 2017). The lack of knowledge on how much the sensors change over time, on the correct time for proper calibration, and on the within-instrument variation may affect the results of validation studies (Gengler, 2019). Furthermore, this study evaluated lactating cows housed in a freestall design. Given that housing systems are associated with effects on dairy cow locomotor activity (Shepley et al., 2020), the ability of the commercial pedometer to measure walking steps in dairy cows housed in different systems, such as pasture, is still uncertain and should be evaluated in future studies.

Although Bland-Altman plots suggested limited bias between the pedometer and observed step counts, numerical differences and distribution pattern of the differences suggested an overestimation of step counts by the pedometer. Similarly, Swartz et al. (2016) demonstrated an overestimation of step counts (118 ± 31 steps/d) by the same pedometer, when compared with visual observations of dairy calves' step activity. In their study (Swartz et al., 2016), a small number of steps measured by the pedometer was observed during the lying periods, suggesting that the device may be recording leg movement as a step while the calf was lying. Although the leg movements of the cows, while they were lying, were not measured in this study, we observed that only 1.5% of the time-blocks recorded by the pedometer as lying behavior had steps counted (data not shown). This suggests that the recording of leg movements as steps is not associated with the overestimation of step counts found in this study. Although our results indicated no overall differences between the visual observations and pedometer measurements, the overestimation of walking step counts by the pedometer (observed to be over 200 steps per day), indicates that the use of this device to represent absolute cow walking activity should be used with caution. In addition, it is still unclear whether the overestimation of steps affects the ability of the sensor to detect estrous events on dairy farms since the thresholds set for these monitors are not only based on absolute step counts but also on the relative increase in steps.

In conclusion, this study demonstrated that the measurements from the commercial pedometer AfiAct II had high agreement with visual observations of step counts in lactating cows whether the steps were measured while walking or in stationary position. However, an overestimation of step counts by the pedometer in comparison to visual observation was found. The commercial pedometer seems to be counting any movement in which the pedometer leg is lifted off the floor, not distinguishing according to body movement of lactating Holstein cows.
